# Illuminating photoreceptors: TGFβ signaling modulates the severeness of retinal degeneration

**DOI:** 10.1038/s41420-025-02685-5

**Published:** 2025-08-15

**Authors:** Aaron Schroers, Andreas Neueder, Isabel Massoudy, Andrea E. Dillinger, Süleyman Ergün, Barbara M. Braunger, Anja Schlecht

**Affiliations:** 1 Institute of Anatomy and Cell Biology, Julius-Maximilians-University Wuerzburg, Wuerzburg, Germany; 2 Center for Molecular Neurobiology Hamburg, University Clinic Hamburg-Eppendorf, Hamburg, Germany; 3 Institute of Neuroanatomy, University Medical Center Hamburg-Eppendorf, Hamburg, Germany

**Keywords:** Visual system, Transcriptomics, Cell death

## Abstract

In various ocular diseases, retinal degeneration (RD) is a clinical symptom that can lead to irreversible vision loss. These diseases include age-related macular degeneration (AMD) and retinitis pigmentosa (RP). Retinal degeneration describes a process during which the retina deteriorates due to the gradual death of photoreceptor cells. Although extensive research has been pursued to identify the underlying pathomechanisms, the precise molecular mechanisms that leads to photoreceptor death remains unclear. In this study, we combined the mouse model of light-induced photoreceptor degeneration with single-cell RNA sequencing to decipher the transcriptional response of degenerating photoreceptor cells. We additionally performed pseudotime analysis of gene expression changes for both the control and light-damaged photoreceptor clusters to analyze the extent of degeneration following a virtual trajectory of severeness. We found a transcriptional heterogeneity of rod photoreceptors in both control and degenerative conditions, and mapped several rod clusters which strongly differ in their transcriptional profile. We defined one of these clusters as the predominant disease-associated rod cluster, containing the most severely damaged rod cells. Pseudotime analysis demonstrated a strong regulation of TGFβ signaling and the RNA-induced silencing complex (RISC) in light-damaged photoreceptors suggesting a pivotal role of these mediators in retinal degeneration.

## Introduction

Millions of people are affected by visual impairment, which strongly impairs many aspects of life, including mobility, independence and mental health [1]. In various ocular diseases, retinal degeneration (RD) is a clinical symptom that can lead to irreversible vision loss. These diseases include age-related macular degeneration (AMD) and retinitis pigmentosa (RP) [2–4]. Retinal degeneration describes a process during which the retina deteriorates due to the gradual death of photoreceptor cells and degeneration of the retinal pigment epithelium (RPE) [5–7]. Although extensive research has been pursued to identify the underlying pathomechanisms, the precise molecular mechanisms that leads to photoreceptor death remains unclear.

To mimic photoreceptor degeneration in mice, several methods including genetic and experimental models have been established and are frequently used. Genetic models are usually characterized by gradual photoreceptor degeneration, with weeks up to months until full percentage of the degeneration is present. As a result, only a small number of cells are at the same stage of degeneration at any given point. This staggered progression hampers the analysis of molecular mechanisms in depth [8, 9].

As an acute model of photoreceptor degeneration, light damage is frequently used and well-established animal model [8]. Exposure to bright, white light simultaneously induces broad and synchronized photoreceptor cell death, which is ideal for investigating the underlying molecular mechanisms in diseases such as RP or AMD [8, 10].

To unravel the molecular mechanism of retinal degeneration, we combined the model of light-induced photoreceptor cell death and single-cell RNA sequencing. Using this approach, we were able to induce a transcriptional response in photoreceptors following light damage and resolve the response by each retinal cell type, in particular in photoreceptor cells, the primary impacted cells. Our data demonstrate that different rod populations with different levels of damage response exist. We propose that these photoreceptor subtypes represent different stages of retinal degeneration. Using pseudotime analysis, we defined distinct gene expression cluster and found that light induced photoreceptor degeneration leads to an altered regulation of TGFβ signaling, highlighting the importance of this signaling pathway for retinal homeostasis and maintenance.

## Results

### Single-cell RNA sequencing identifies distinct populations of photoreceptors

**Fig. 1:**
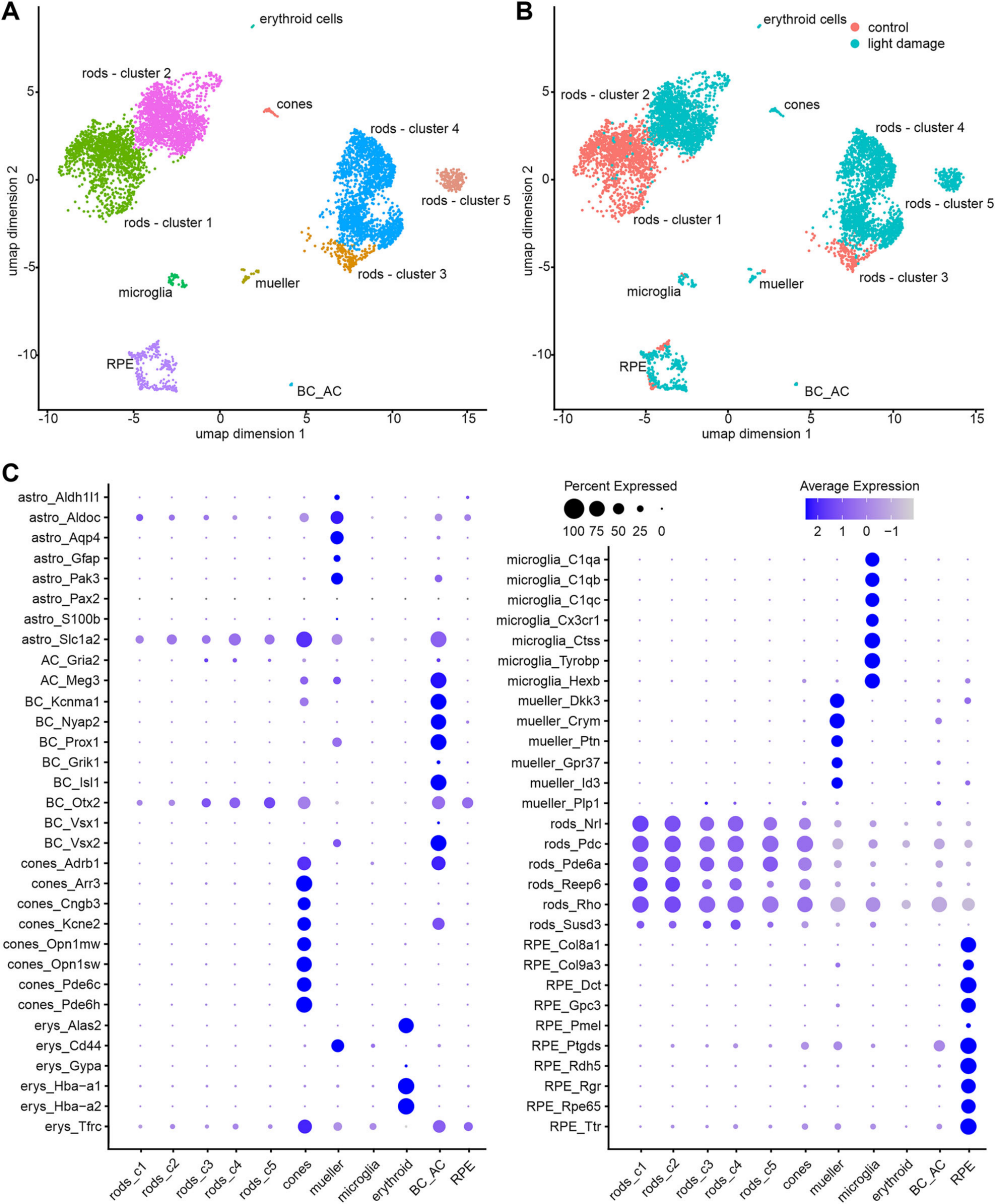
Single-cell RNAseq of control and light damaged retinae. **A**. Single-cell RNA expression data were used to cluster cells and assign cell types as described in the methods section. Uniform manifold approximation and projection (umap) dimensions 1 and 2 for each cell type are shown. **B**. Light damage led to a distinct separation of the rod clusters in comparison to control conditions (see rod—cluster 1 and 2 and rod cluster 4 and 5). In addition, a new rod cluster was evident under light damage conditions (rod cluster 5). **C** Expression of cell-type markers (columns) vs. identified clusters (rows) as shown in (**A**). The percentage of cells in each cluster is represented by the size of the circle and average expression level by color depth.

**Fig. 2:**
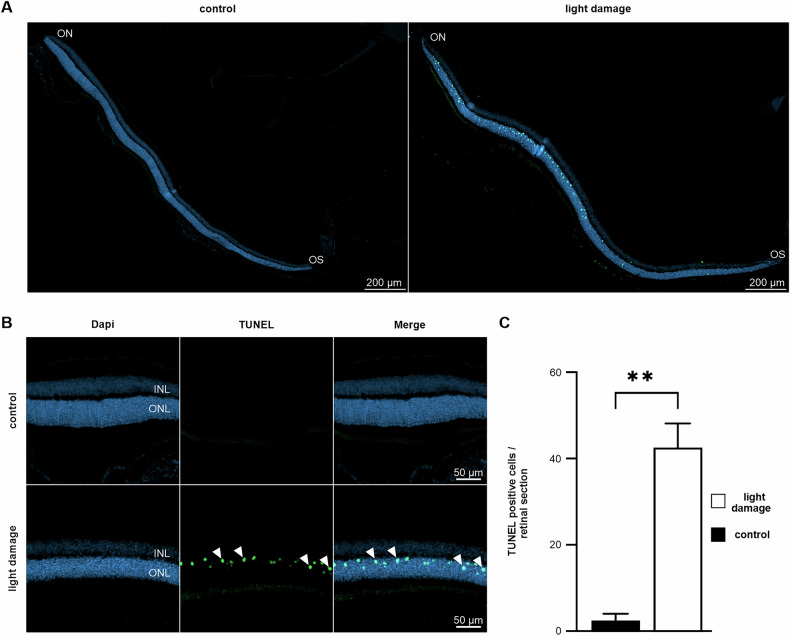
Cell death following light-induced photoreceptor degeneration. Mid-horizontal sections of TdT-mediated dUTP-biotin nick end (TUNEL)-labeled (green, arrowheads) control and 6 h following light damage retinae (**A**). Detailed magnification (**B**) Cell nuclei were stained with DAPI (blue). **C** Total number of TUNEL-positive cells per section stretching through the optic nerve head (control *n* = 2 mean: 2.5 ± 1.5 (SEM); light damage *n* = 5 mean: 42.6 ± 5.5 (SEM); *P* = 0.0075; *t*-test. OS ora serrata, ON optic nerve, INL inner nuclear layer, ONL outer nuclear layer.

To study the transcriptome of individual cells during early phase of photoreceptor degeneration, we collected retinae from dark-adapted (controls) and light-exposed mice for single cell RNA sequencing analyses (Fig. 1). Prior to RNA sequencing we confirmed the successful induction of light induced photoreceptor degeneration using TUNEL assay as provided in Fig. 2. For details about the single cell isolation and sequencing procedures please see the methods section. Briefly, we isolated whole cells from control and light-damaged retinae, which allowed us to study the whole cellular transcriptome. Cells were barcoded using the 10x Genomics v3.1 chemistry and sequenced on a Illumina NovaSeq 6000. Using a state-of-the-art bioinformatics pipeline combining the Alevin-Fry and Seurat frameworks together with a machine-learning-based cell clustering algorithm allowed us to identify 11 different cell types (Fig. 1A). Cell types overlapped largely for control and light-damaged retinae, with the exception of rods, which showed distinct clusters (Fig. 1B, e.g., rod cluster 1 and 2). The expression of known marker genes [4, 11–13] for each cluster demonstrated correct assignment of cell types and very good separation of cell types (Fig. 1C). The expression levels of an extended list of marker genes across the clusters is shown in Supplementary Fig. 1. In summary, we identified 5 distinct rod clusters and one cluster each for cones, muller cells, microglia, retinal pigmented epithelial cells and cells expressing markers for both, amacrine and bipolar cells.

As mentioned above, most clusters contained cells from retinae of mice with and without light damage. However, we identified clusters of rods that almost exclusively contained cells with light damage (rod cluster 2, 96.9%; rod cluster 4, 99.1%; rod cluster 5, 100%), as well as clusters that predominantly contained control cells (rod cluster 1, 97.7% and rod cluster 3, 94.3%) (Fig. 1A, B), strongly suggesting that control and light-damaged rods differ in their transcriptional profile and distinct populations of rods with different levels of stress response exist, in particular in the light damaged retina (Fig. 1B).

### Gene ontology enrichment analysis of light damage-affected cell populations

**Fig. 3:**
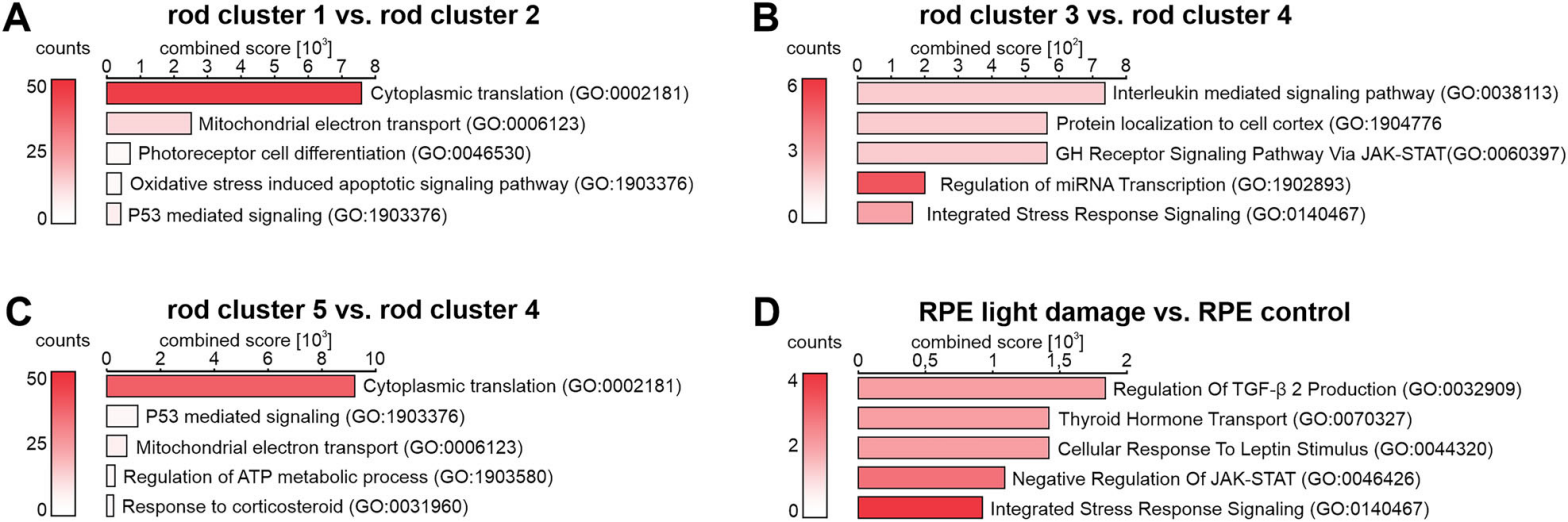
Gene Ontology enrichment analysis of UMAP cluster comparisons. Inter-cluster comparisons between rod cluster 1 and 2 (**A**), rod cluster 3 and 4 (**B**) and rod cluster 5 and 4 (**C**). **D** Gene Ontology enrichment analysis of RPE cells from the light-damaged retinae and controls. For details regarding GO enrichment analysis, see “Methods” section. The number of genes found for each GO term is represented by the color of the graphs.

Since photoreceptor degeneration is the primary event happening during light-induced damage, we specifically focused on treatment effects on rod clusters and on the retinal pigmented epithelium as a supporting cell population. Umap dimensions suggested that rod clusters 1 and 2, as well as 3 and 4 formed related pairs of control and light damaged rod sub-populations. Rod cluster 5 was specific to light damage (Fig. 1B). Therefore, we compared rod cluster 1 (control) with its neighboring rod cluster 2 (light damage) and found 403 differentially expressed genes (DEG, Benjamini-Hochberg adjusted *P*adj-value < 10^-6^, Supplementary Table 1), with phosphodiesterase 6 G (*Pde6g)* being the most dysregulated gene (adj. p: 9.05E-232). *Pde6g* is known to be an early-phase-regulated gene during photoreceptor degeneration [4]. To further determine which biological processes the DEG contribute to, we performed a functional gene ontology (GO) cluster analysis (Fig. 3A). Among the resulting top five GO terms were cytoplasmic translation (GO:0002181), mitochondrial electron transport (GO:0006123), oxidative stress induced apoptotic signaling pathway (GO:1903376) and enhanced p53 mediated signaling (GO:1903376). All of them suggesting the induction of an early stress response and beginning apoptosis [14–19]. Consistently, rod cluster 1 contained the majority of undamaged rod cells (control) and only few control rod cells (cluster 3) were shifted towards the other large clusters of damaged rods (clusters 4 and 5) (Fig. 1B).

Next, we compared rod cluster 3 (control) to its neighboring rod cluster 4 (light damage) (Figs. 1A and 3B) and identified 107 DEGs (*P*adj < 10^−6^, Supplementary Table 1). These genes contributed to processes such as Interleukin mediated signaling pathway (GO:0038113), growth hormone receptor signaling via JAK-STAT (GO:0060397) and integrated stress response (GO:0140467). Both, interleukin signaling, as well as JAK-STAT pathway are known to be implicated in late stages of photoreceptor degeneration and/or retinal degeneration [20–23].

Finally, we analyzed the isolated cluster of rods (cluster 5), which appeared separately from rod clusters 1–4 in the UMAP analysis. This cluster consisted exclusively of light-damaged photoreceptors and clearly separated from the other light-damaged rod populations 2 and 4 in UMAP dimension 1 (Fig. 1A, B). The top 5 significantly regulated factors in rod cluster 5 were *Malat1, Egr1, Cst3, Unc13b, H3f3a* (Supplementary Table 2). To identify the transcriptional changes, which were responsible for the further shift of these rod cells as compared to the already well-affected population in rod cluster 4, we compared rod cluster 5 with rod cluster 4. Corroborating the implied trajectory of increased damage, we found clear evidence of induction of apoptosis, stress response and alterations in the regulation of energy supply (Fig. 3C, Supplementary Table 1).

The retinal pigment epithelium is a neighboring and supporting cell population to the rods but also functionally involved in processes such as the visual cycle. To analyze whether the RPE responds cell-autonomously to light-induced photoreceptor damage, we compared the control cells within the RPE cell cluster with those from light-damaged retinae (Fig. 1A, B). Dysregulation analysis showed 38 DEGs (Supplementary Table 1) and several affected pathways by gene ontology analysis (Fig. 3D). The top dysregulated pathway following light damage was regulation of TGF-β 2 production (Fig. 3D), thus supporting the concept that TGFβ signaling is involved in photoreceptor maintenance and survival [24–26].

### Pseudotime analysis

**Fig. 4:**
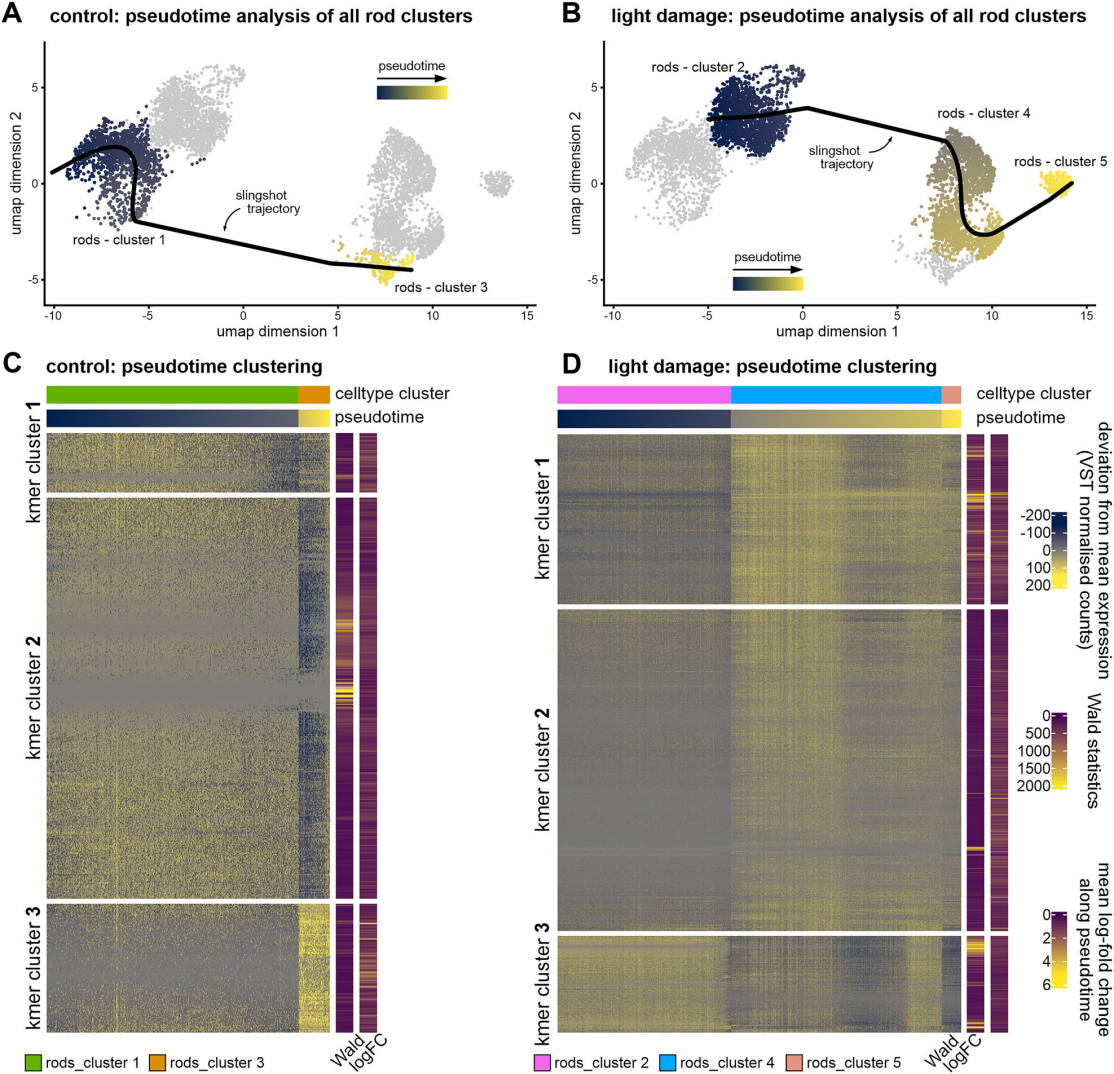
Pseudotime analysis of control and light-damaged rod clusters. **A**, **B** Pseudotime analysis of rod clusters using slingshot analysis. Slingshot trajectories were computed across rod clusters 1 and 3 for the control condition and rod clusters 2, 4 and 5 for the light damage condition. **C**, **D**. We only used genes for unsupervised clustering analysis that had an FDR-adjusted GAM *P*-value of 0 in the slingshot analysis (see methods). The expression of these genes (VST normalized counts) was used to obtain 3 clusters of co-regulated genes along the pseudotime trajectory for control (**C**) and light damage (**D**) conditions. Wald test statistics (a measure of how well the expression of a gene follows the pseudotime trajectory) and log-fold change of gene expression along the pseudotime trajectory are shown on the right side of the heatmaps.

**Fig. 5:**
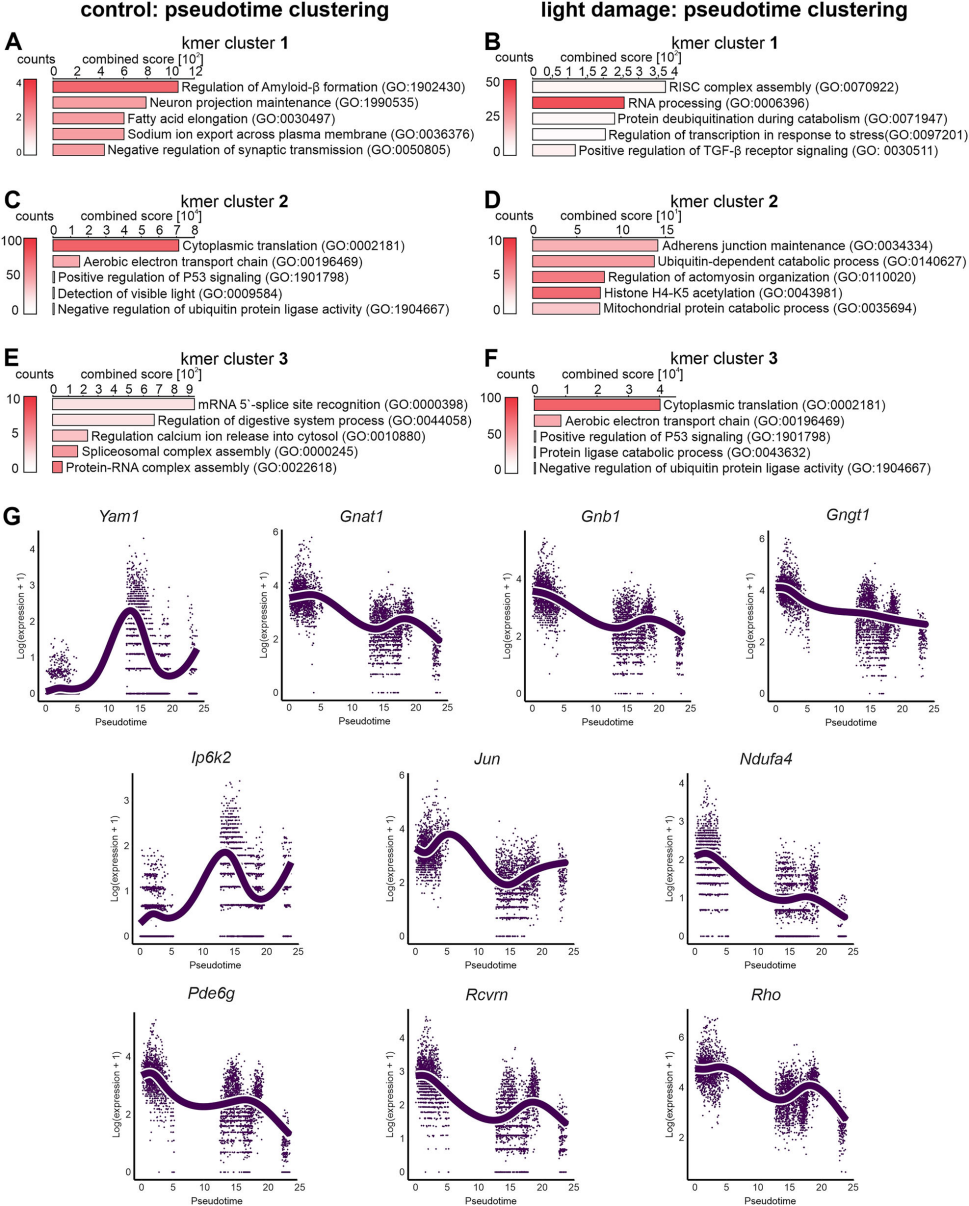
Gene ontology enrichment analysis of pseudotime analysis and visualization of dysregulated genes. **A**–**F** Gene ontology enrichment analysis of k-mer clusters from pseudotime analysis. For details regarding GO enrichment analysis, see “Methods” section. The number of genes corresponding to each GO term is represented by the color of the graphs. **G** Visualization of the most significantly dysregulated genes following pseudotime. Graphs represent top 10 genes with expression pattern corresponding most closely to the pseudotime under light damage conditions.

As described above, the UMAP analysis demonstrated that different rod populations exist under both control and light damage conditions. Since the gene ontology analysis indicated that these might represent different stages of degeneration, we performed a pseudotime analysis of gene expression changes for both the control and light damage clusters to analyze the extent of degeneration following a virtual trajectory of severeness. We used the slingshot package, to create one-dimensional trajectories and to link cells across various stages, also known as “pseudotime.” Slingshot trajectories were computed across rod clusters 1 and 3 for the control condition and rod clusters 2, 4 and 5 for the light damage condition (Fig. 4A, B). In the next step, we defined genes whose expression profile most closely follows the slingshot trajectory. To model the associations between gene expression and pseudotime, a general additive model (GAM) using a negative binomial noise distribution was fitted. After calculating statistically significant gene expression differences, we only considered genes that had an adjusted *P*-value of 0 and therefore statistically perfectly followed the pseudotime for further analysis. This resulted in 526 genes for the control condition and 3317 genes for the light damage condition, which were significantly associated with pseudotime across the rod clusters (Supplementary Table 3). These genes were subsequently clustered by an unsupervised clustering strategy (k-mer = 3) (Fig. 4C, D). For control rods, 3 k-mer clusters were defined as follows: k-mer Cluster [1] included 53 genes and showed a high expression during the early course of the pseudotime analysis with a mild decrease over time (Figs. 4C and 5A). K-mer cluster 2 included 358 genes with a high gene expression during early stages and a strong decrease at the end of pseudotime (Fig. 4C). This gene expression profile displays a high similarity to k-mer cluster 3 (545 genes) from light damage condition (Fig. 4D). This is particularly evident in the GO enrichment analysis of these two k-mer clusters (Fig. 5C, F), which contain biological processes indicative of increased cellular stress. Since this applies to both the control and the light damage situation, we assume that these are effects caused by the processing and separation of the cells in preparation for sequencing [27–29]. K-mer cluster 3 of the control condition contained 115 genes, which were characterized by a rather low expression during early pseudotime and a high expression in late stages. These genes contributed predominantly to processes associated with splicing (Fig. 5E).

Light damage rod cells exhibited two additional cluster, k-mer cluster 1 and 2 (Fig. 4D). 1813 genes in k-mer cluster 2 (Fig. 4D) follow a pattern in which they initially show low expression levels, followed by a gradual increase, and subsequently decline. Genes in this cluster participate to processes such as histone acetylation pointing towards photoreceptor degeneration (Fig. 5D) [30, 31]. K-mer cluster 1 contains 959 genes (Fig. 4D), displaying a low expression at early phases, followed by a continuous increase in gene expression levels. These genes, such as e.g. *Ago 2*, were strongly associated with the RNA-induced silencing complex (RISC). Several studies describe that malfunction of RISC leads to retinal degeneration [32, 33]. Furthermore, we found a strong enrichment of the TGFβ signaling pathway which might again support the concept that TGFβ signaling contributes to maintenance and neuroprotection during photoreceptor cell death (Fig. 5B) [24–26].

Of note, we also determined the top 10 genes whose expression pattern corresponded most closely to the pseudotime under light damage conditions. (Fig. 4B). Six out of these 10 genes (*Gnat1: G Protein Subunit Alpha Transducin 1, Gnb1: G Protein Subunit Beta 1, Gngt1: G Protein Subunit Gamma Transducin 1, Rho: Rhodopsin, Pde6g, Rcvrn: Recoverin*) are mediators of phototransduction and were strongly downregulated following pseudotime (Fig. 5G). This is of particular interest as these mediators are known to be downregulated also in the course of genetic, progressive photoreceptor degeneration [4], confirming our hypothesis that the photoreceptors in cluster 5 represent the most severely damaged cell population.

## Discussion

The present study provides novel insights into the molecular mechanisms underlying retinal degeneration by utilizing single-cell RNA sequencing in a mouse model of light-induced photoreceptor degeneration. Our findings highlight the transcriptional heterogeneity of rod photoreceptors in both control and degenerative conditions, offering a deeper understanding of the pathways involved in photoreceptor degeneration in diseases such as retinitis pigmentosa or AMD. We identified several rod clusters that strongly differ in their transcriptional profile. Neuronal damage led to the appearance of additional rod populations with cells exhibiting differential responsess to the insult. By applying trajectory pseudotime we discovered a strong regulation of TGFβ signaling and the RISC complex in light-damaged rods suggesting a pivotal role of these mediators in photoreceptor degeneration.

The identification of rod cluster 5, representing the distinct rod subpopulation of most severely light-damaged photoreceptors, provides the opportunity to gain critical insights into molecular changes occurring during advanced photoreceptor degeneration. Among others we found a strong upregulation of *Egr1* and *Malat1* in cluster 5 rods. *Egr1* is a transcription factor involved in cellular stress responses and has already been reported to play a key role in mouse models of photoreceptor degeneration [4, 34–36]. In accordance with our findings, studies report a significant upregulation of *Egr1* during photoreceptor degeneration in several mouse models [4, 37, 38]. Various signaling cascades regulate *Egr1* as a downstream effector, including those associated with (retinal) neuroprotection, such as P38 MAPK singlaing [35, 36, 39]. Transcription of the *Egr1* gene is regulated by the mitogen-activated protein kinase (MAPK) signaling pathway [34, 40, 41]. Inhibition of either EGR1 itself or MAPK signaling pathway results in a reduction of photoreceptor degeneration [34, 42, 43].

During the last years, long non-coding RNAs (lncRNAs) have also been implicated to play regulatory roles in neurodegeneration. Unlike protein-coding RNAs, lncRNAs modulate gene expression through epigenetic, transcriptional, and post-transcriptional mechanisms [44]. In the eye, several lncRNAs have been associated with retinal diseases, influencing photoreceptor survival and degeneration [45]. We found Malat1 to be one of the most upregulated genes in cluster 5 rods, suggesting a pivotal role for *Malat1* in advanced photoreceptor degeneration. This is in line with previous results from Yao et al. and Zhang et al. who describe a role for Malat1 in neuroprotection and cellular stress responses in retinal degeneration [46, 47]. This is of particular interest as silencing Malat1 leads to a mitigated effect in photoreceptor degeneration [47].

Besides the regulation of *Egr1* and *Malat1* in the dominant disease-associated clusters of photoreceptors, we detected a strong association of the RNA-induced silencing complex (RISC) with the progression of photoreceptor degeneration by applying trajectory pseudotime. RISC is a key effector of RNA interference, primarily functioning in post-transcriptional gene silencing by mediating the cleavage or translational repression of target mRNAs [48]. This complex consists of Argonaute proteins such as Ago2 and small RNAs, such as microRNAs (miRNAs) or small interfering RNAs (siRNAs), which guide the RISC to specific mRNA targets [49]. In retinal homeostasis, RISC plays a crucial role by regulating gene expression networks involved in cell survival, metabolism, and response to stress [50–52]. miRNAs processed within RISC contribute to retinal development and maintenance, ensuring proper function of retinal cells, including photoreceptors, by controlling apoptosis and neuroprotection-related pathways [32, 53, 54]. During retinal degeneration, dysregulation of the RISC complex and altered miRNA expression can result in increased cell death and inflammation [32, 55, 56]. For instance, downregulation of neuroprotective miRNAs such as miR-21 exacerbates degenerative processes, whereas overexpression of other miRNAs may counteract retinal damage [57–60]. Specifically, in photoreceptor degeneration, the RISC complex modulates stress response genes and apoptotic regulators, significantly impacting cell survival [32, 61, 62]. Several miRNAs, as well as Argonaute proteins are crucial for photoreceptor maintenance, and their loss has been associated with increased susceptibility to degeneration [33, 59, 63, 64]. Thus, targeting the RISC may represent a potential therapeutic avenue for mitigating photoreceptor loss in retinal degeneration.

Furthermore, we found a strong association between pseudotime analysis photoreceptor degeneration and TGFβ signaling. Canonical TGFβ signaling plays a fundamental role in retinal development and homeostasis by regulating cell growth, migration, proliferation and death [26, 65–71]. In a disease context, the TGFβ pathway has been associated with neovascular eye diseases such as age-related macular degeneration, diabetic retinopathy and retinopathy of prematurity in both, mice and humans [72–79]. However, there is emerging evidence, that TGFβ signaling also possesses neuroprotective properties. Initial evidence for this hypothesis was obtained by inactivating the signaling pathway in the adult eye via application of soluble endoglin. This resulted in neuronal apoptosis [68]. As demonstrated by our group, this effect is not limited to the adult age. We were able to show that the absence of the TGFβ signaling pathway in retinal neurons and muller cells also leads to an increased apoptosis rate during embryonic development [26]. These results indicate that the signaling pathway plays a fundamental role for retinal neurons both in the development of the neuroretina and in the maintenance of neuronal homeostasis in the eye. Even more interesting, we found that TGFBR2 is upregulated in neurons and Müller cells in VPP mice, a genetic mouse model for retinitis pigmentosa [24]. Inhibiting TGFBR2 in these cells resulted in drastically exacerbated photoreceptor degeneration in VPP-induced photoreceptor degeneration [25], which lead to the conclusion that TGFβ signaling in retinal neurons and Müller cells exhibits a neuroprotective effect. In the present study, we also observed a strong association between TGFβ signaling and disease progression during light-induced photoreceptor degeneration. These data suggest that the regulation of TGF-β signaling is a shared key mechanism in retinal degeneration and may be relevant across different types of diseases u mice and potentially in humans.

## Conclusion

In summary, this study shows a transcriptional heterogeneity of rod photoreceptors particularly in light-induced degenerative conditions. We defined a predominant disease-associated rod cluster, containing the most severely damaged rod cells. Pseudotime analysis of gene expression changes displayed a virtual trajectory of severeness concomitant with a strong regulation of TGFβ signaling in light-damaged photoreceptor cells. Our findings suggest that the regulation of TGF-β signaling is a key mechanism in retinal degeneration. This is of particular interest given the current lack of curative treatments for retinal degeneration. Consequently, cell-type-specific targeting of the TGFβ signaling pathway could represent a promising and innovative therapeutic target for patients suffering from retinal degeneration in the future.

## Materials and methods

### Mice

All procedures conformed to the tenets of the National Institutes of Health Guidelines on the Care and Use of Animals in Research, the EU Directive 2010/63/E. Mice carrying two floxed *Tgfbr2* alleles [80], thus presenting functional wildtype mice, were bread on a Balb-C/CD1 background and kept in cyclic light (12 h on/12 h off, lights on at 7 am, light intensity approx. 400 lx). Both sexes were used for experiments and all mice were homozygous for the L450 variant of RPE65.

### Light damage

Experimental 6–9 week-old mice were dark-adapted in cyclic dim light (<50 lux) followed by a period of complete darkness for 18 h prior to light damage. Mice were then placed in reflective cages and exposed to diffuse cool, white fluorescent light coming from the top of the cage with an intensity of 5000 lx for 30 min. After light exposure, mice were allowed to recover for 6 h in dim light, and then sacrificed. Light damage experiments were always performed in the early morning. Mice that were dark-adapted but did not receive light damage served as control mice.

### TUNEL labeling and fluorescence microscopy

Mice were euthanized by CO_2_ exposure followed by cardiac perfusion with 10 ml ice-cold 0,1 M phosphate buffer. The eyes were marked with a branding at the superior limbus. Eyes were enucleated, followed by fixation in 4% PFA. The branding was marked using a short, thin metal needle and the eyes were embedded in paraffin according to standard protocols [72, 81]. Terminal desoxynucleotidyl transferase-mediated dUTP-biotin nick end labeling (TUNEL, Dead-EndTM Fluorometric TUNEL System, Promega, #G3250) was performed on sections that were cut along the mid-horizontal plane (in nasal-temporal orientation) stretching through the optic nerve head (ONH) according to our previously published protocol [26, 81]. To quantify the number of apoptotic cells, images of the whole retina were taken using an Axiovert 135 microscope (Zeiss). TUNEL-positive cells were counted manually.

### Single cell isolation

Mice were euthanized by CO_2_ exposure followed by cardiac perfusion with 10 ml ice-cold PBS. Eyes were immediately dissected to isolate the retinae. Retinae were dissected in 500 µl 0.25 mM DMEM/HEPES containing 120 U/ml papain (Carl Roth, #9001-73-4), 225 U/ml collagenase IV (Worthington, #LS004188) and 20 U/ml DNase I (Worthington, #LS006344). Prior to digestion at 8 °C for 45 min, each sample was mechanically triturated by pipetting. After digestion, cell suspensions were filtered through a 50 µm cell strainer. Retinae from 3 to 5 mice were pooled to obtain sufficient cell numbers. To remove dead cells and debris, cell suspension was incubated with magnetic beads (DeadCell Removal Kit, Milteny, # 130-090-101). Cells were sorted through MS Columns (Milteny, # 130-042-201) installed in an OctoMacs magnet (Milteny, # 130-042-109) according to manufactures protocol. Intact and viable single cells were collected and resuspended in ultra-pure BSA (1:1000, Thermo Fisher, #AM2616).

Prior to processing for scRNAseq, the single-cell suspensions were again filtered through a 40 µm strainer. Cell concentrations were assessed using an automated cell counter. The concentration for each sample was adjusted to approximately 1,000 cells/µL resulting in approx. 10,000 cells per reaction.

### Single-cell RNAseq

We used the 3’ 10x Genomics Chromium Single Cell Gene Expression protocol (v3.1) to capture and barcode mRNA transcripts from the single cell suspensions. Briefly, the cell suspensions, together with barcoded gel beads, master mix, and partitioning oil, was loaded on a 10x Genomics Chromium Next GEM chip. Using a Chromium Controller, the cells were partitioned into “gel bead in emulsions” (GEMs). Within each GEM, reverse transcription was followed by barcoding of transcripts with a unique 10x barcode and unique molecular identifiers (UMIs). The cDNA was purified using Silane dynabeads followed by PCR amplification. The amplified cDNA was fragmented, end repaired and A-tailed to create compatible ends for adapter ligation. Sequencing adapters (P5 and P7) were ligated to the cDNA fragments, and a sample index was added during a final round of PCR amplification, enabling multiplexing of samples in a single sequencing run. The constructed libraries were purified using AMPure XP beads before cDNA concentration and fragment size distribution were assessed using a Qubit Fluorometer and an Agilent Bioanalyzer. Libraries were pooled in equimolar ratios for sequencing.

Libraries were sequenced on an Illumina NovaSeq 6000 platform using the 100 cycle S2 reagent kit in paired-end mode. Sequencing parameters were set to 28 bp for Read 1 (cell barcode and UMI), 8 bp for the i7 index (sample index), and 91 bp for Read 2 (cDNA insert). We aimed for a sequencing depth of approximately 50,000 reads per cell. Sequencing data were demultiplexing with the 10x Genomics Cell Ranger software pipeline (v.7.0.1).

### Bioinformatics

Reads were mapped and quantified using the Alevin-Fry framework (alevin-fry v0.8.1, salmon v1.10.1, pyroe v0.9.2, python v3.9.16) [82]. To this end, we created a splici index using the 10x genomics mouse mm10 reference data (refdata-gex-mm10-2020-A) with a read length of 90. Reads were mapped with the salmon alevin command using ISR as the library type and chromiumV3 as the library type. We used sketch mode to generate a rad file by pseudoalignment with structural constraints. Next, we generated a barcode permit list using the 10x Genomics barcodes whitelist (3M-february-2018.txt). Additionally, we filtered reads that mapped to the reverse complement strand of transcripts by specifying -d fw. Lastly, UMIs per-gene and per-cell were quantified using the ‘cr like’ strategy.

All subsequent analyses were conducted in R v4.4.1 [83]. For quality control (QC) and filtering we used the singleCellTK package [84]. First, we filtered droplets by running the runEmptyDrops() and runBarcodeRankDrops() on the single-cell datasets. We removed all droplets with a BarcodeRank_Inflection of 1, all droplets with non-computable FDR and all droplets with FDR < 0.01 for the probability to be an empty droplet. Next, we ran per-cell QC using the runCellQC() function using the ‘QCMetrics’, ‘scDblFinder’ and ‘decontX’ algorithms. Final droplets were filtered using a mitochondrial percentage of less than 10% and a scDblFinder call of being a ‘singlet’. Droplets with large ambient RNA decontamination (≥ 0.5) as determined by decontX were also removed (Supplementary Fig. 2).

Filtered datasets were imported into Seurat v5.1.0 [85]. Next, we tested 5 different clustering algorithms (CCA, RPCA, Harmony, FastMNN and SCVI) and analyzed the dimensionality of the clustered datasets using elbow plots. A dimensionality of 50 was used for all subsequent analyses. Additionally, we tested three algorithms (CCA, RPCA, Harmony) on SCT v2 transformed data. Next, we used a resolution of 0.3 and a list with specific markers for each cell type of interest to assess the effectiveness of the different clustering strategies. We found that the SCVI clustering strategy gave the most distinct clusters with very little marker spillover. SCVI (single-cell variational inference tools [86]) is a package for probabilistic modeling of single-cell omics data utilizing PyTorch and AnnData. We trained models with a single layer and up to 50 latents (dimensions) for 50–400 epochs. The best performing model was trained with 1 layer, 50 latents and ‘number of RNA counts per cell’ and ‘mitochondrial percentage’ as continuous covariates for 200 epochs. Learning rate and other parameters were kept at their default values. To assign final cell types, we clustered cells by the trained SCVI model in Seurat with a resolution of 200 (Supplementary Fig. 3). This resulted in very fragmented cell clusters, which we merged again with guidance of the coordinates of the UMAP dimensions 1 and 2. The expression of specific markers for each cell type is shown in Supplementary Fig. 1. The expression of specific markers across UMAP dimensions 1 and 2 is shown in Supplementary Fig. 4. Dysregulated genes were calculated using the FindMarkers() function in Seurat either between cell type clusters or between a specific cluster and all other cells. Intra- and intercluster comparisons are provided in Supplementary Table 1 and 4.

Pseudotime analysis was carried out using the slingshot v2.12.0 package [87]. We used RNA counts as input together with the computed SCVI UMAPs. A general additive model (GAM) using a negative binomial noise distribution was then fitted to model the associations between gene expression and pseudotime. Statistical significant gene expression differences were calculated using the associationTest() function and *P*-values were corrected using the ‘FDR’ method. To address the overfitting and potentially large number of false positive associations by using cells as samples, we only considered genes that had an adjusted *P*-value of 0 for further analysis. This resulted in 526 genes for the control condition and 3317 genes for the light damage condition, which were significantly (*P*_adj_ = 0) associated with pseudotime across the rod clusters. These genes were subsequently clustered by an unsupervised clustering strategy (k-mer = 3) across pseudotime using the ComplexHeatmap v2.20.0 package [88]. The scripts for bioinformatics analyses are available upon reasonable request.

Ontology analysis was carried out using the Enrichr website (https://maayanlab.cloud/Enrichr, accessed on 2nd September 2024). Only statistically differently expressed genes with were included in the analysis. We used adjusted *P*-value cutoffs of *P*_adj._ < 10^-6^ (Fig. 3) and *P*_adj._ = 0 (Fig. 5). These stringent cutoffs were used to mitigate the problem of negatively inflated *P*-values due to each cell being treated as a biological replicate (see also above). GO biological process (2023) terms were sorted according to the combined score. All terms with an adjusted *P*-value > 0.05 were excluded from the analysis. The five most relevant, non-redundant enriched terms are shown.

### Statistical analysis

Statistical analysis was performed using GraphPad Prism (GraphPad Software, Version 6.0, La Jolla, CA, USA). For comparison of two groups (TUNEL labeling) students t-test was used. Data is shown as mean ± SEM. Sample size estimation was informed by prior experience with the specific mouse model employed in this study. No randomization was used. Quantification of TUNEL+ cells was performed blinded for both groups. Differences were considered to be statistically significant for *P*-values < 0.05.

## Supplementary information

Supplementary Legends

Supplementary Figure 1

Supplementary Figure 2

Supplementary Figure 3

Supplementary Figure 4

Supplementary Table1

Supplementary Table2

Supplementary Table3

Supplementary Table4

## Data Availability

RNA Sequencing Data (GSE291654) is accessible from https://www.ncbi.nlm.nih.gov/geo/query/acc.cgi?acc=GSE291654.
