# Supplementary material for: Illuminating photoreceptors: TGFβ signaling modulates the severeness of retinal degeneration
Source: Cell Death Discov. 2025 Aug 15;11:384. doi: 10.1038/s41420-025-02685-5 (PMC12356984; doi:10.1038/s41420-025-02685-5)
Supplement: Supplementary file 1 — Supplementary Legends [file 41420_2025_2685_MOESM1_ESM.docx]

# Supplementary Figure Legends

**Supplementary Figure 1:** **Expression of cell type specific markers for each population.**

Expression of cell-type markers (rows) vs. identified clusters (columns) as shown in Figure 1A. The percentage of cells in each cluster is represented by the size of the circle and average expression level by color depth.

**Supplementary Figure 2: Single cell RNAseq data quality control**

After mapping and quantification of RNAs as described in the methods section, raw data were analyzed with the singleCellTK package. Specifically, total counts (**A**), total features (**B**) and mitochondrial reads (**C**) per cell were counted. Both conditions showed very comparable results. **D** To account for ambient RNA contamination, we computed ambient RNA with decontX and used the contamination score to remove droplets with very high ambient RNA contamination ( ≥ 0.5). The decontX corrected counts were used for further analysis. **E** Overview of cell numbers before (raw data) and after filtering as described in the methods section.

**Supplementary Figure 3: Single cell RNAseq – SCVI clustering**

We used single-cell variational inference tools (SCVI) to cluster our cells (see also methods section). Training (**A**) and validation (**B**) elbow plots showed good convergence at about 200 epochs in the model with 50 latents (see C, 50 latents). **C** We trained the SCVI models with up to 50 latents and computed the clustering every 5 latents. UMAP dimension 1 is shown at the x-axis and UMAP dimension 2 is shown at the y-axis. We used a resolution of 0.3 for the latent screen. We observed good separation of cell clusters starting at about 20 latents. After about 40 latents, changes to the clustering were very small. The final model is identical to what is shown at 50 latents (see also main figure).

**Supplementary Figure 4:** Gene expression of specific markers across UMAP dimensions 1 and 2 for (A) rods, (B) amacrine cells, (C) bipolar cells, (D) astrocytes, (E) cones, (F) endothelial cells, (G) erythrocytes, (H) microglia, (I) Mueller cells, (K) RPE cells.

# Supplementary Table Legends

**Supplementary Table 1:** DEGs from intercluster comparisons between rod_c2 and rod_c1, rod_c4 and rod_c3, rod_c5 and rod_c4 and between mueller, microglia and RPE cells from the lightdamage and control group.

**Supplementary Table 2:** Maker Genes from UMAP clustered rods (rods_c1-rods_c5), mueller, microglia and RPE cells. For every cell population except rods separated by experimental group.

**Supplementary Table 3:** Genes, which were significantly (*P*_adj_ = 0) associated with pseudotime across the rod clusters, clustered by an unsupervised clustering strategy (k-mer = 3)

**Supplementary Table 4:** DEGs from intercluster comparisons between rod_c1 and rod_c3, rod_c1 and rod_c4, rod_c1 and rod_c5, rod_c2 and rod_c4, rod_c2 and rod_c5.
