# Supplementary figures and images for: Illuminating photoreceptors: TGFβ signaling modulates the severeness of retinal degeneration
Source: Cell Death Discov. 2025 Aug 15;11:384. doi: 10.1038/s41420-025-02685-5 (PMC12356984; doi:10.1038/s41420-025-02685-5)

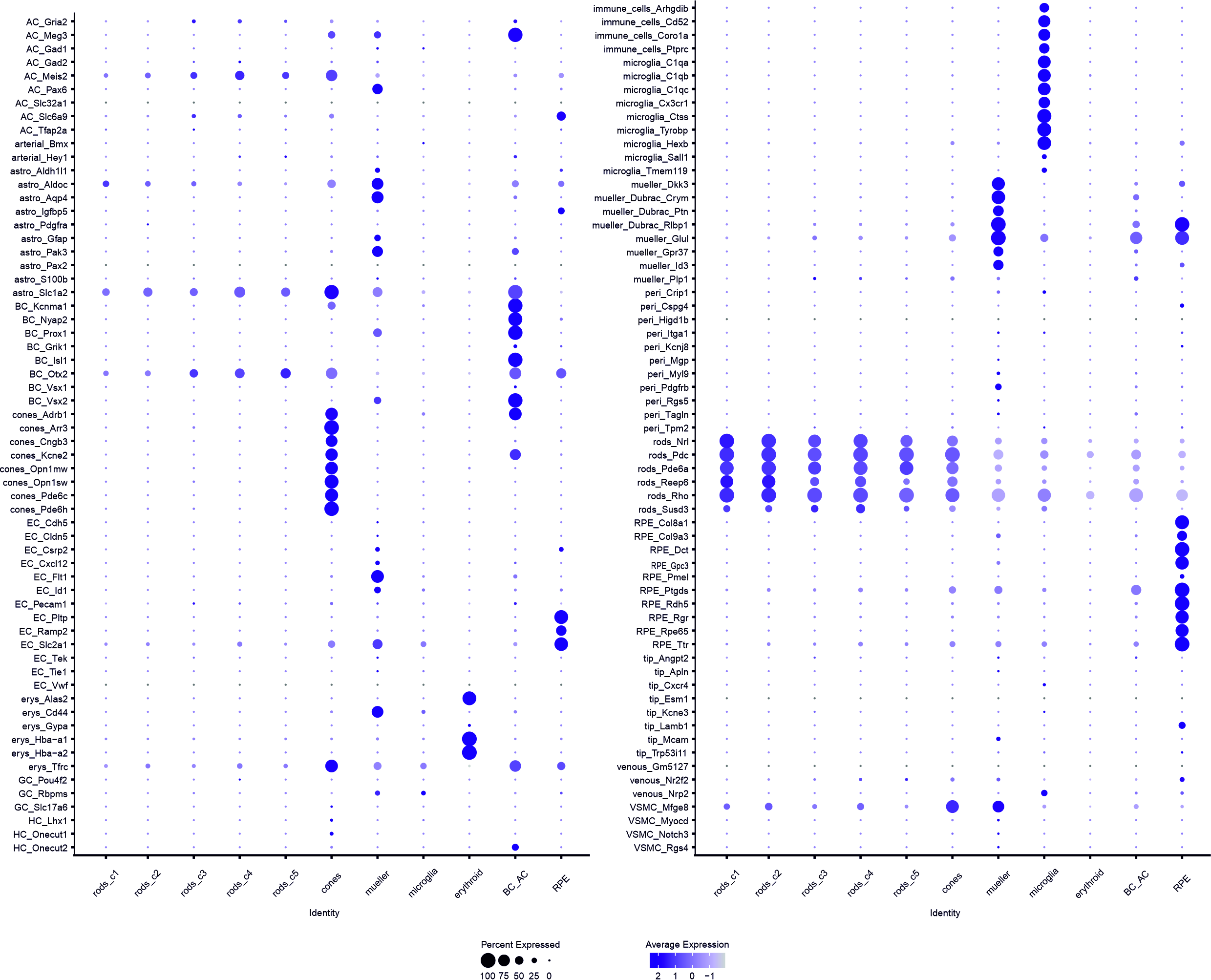

Supplement: Supplementary file 2 — Supplementary Figure 1 [file 41420_2025_2685_MOESM2_ESM.tif]

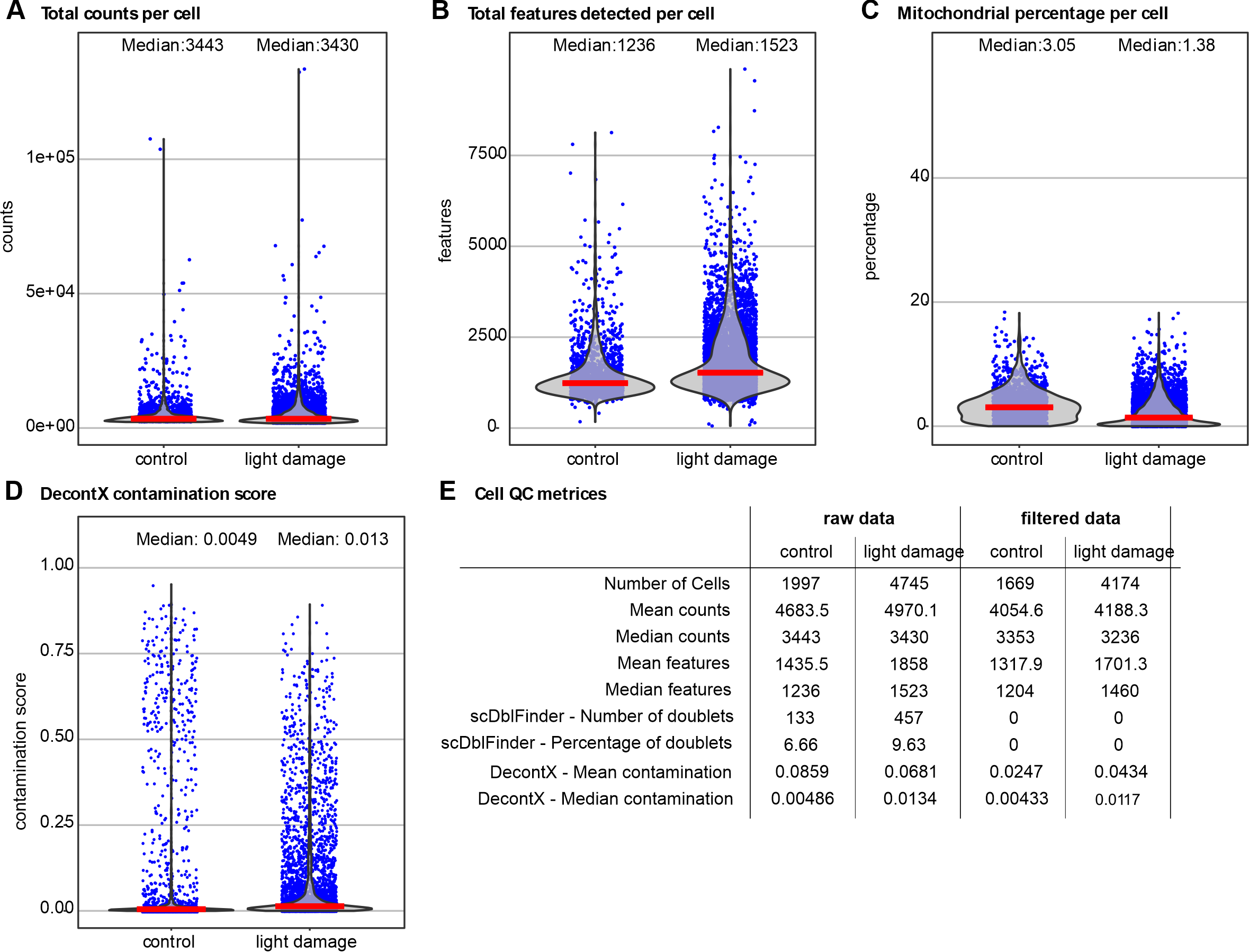

Supplement: Supplementary file 3 — Supplementary Figure 2 [file 41420_2025_2685_MOESM3_ESM.tif]

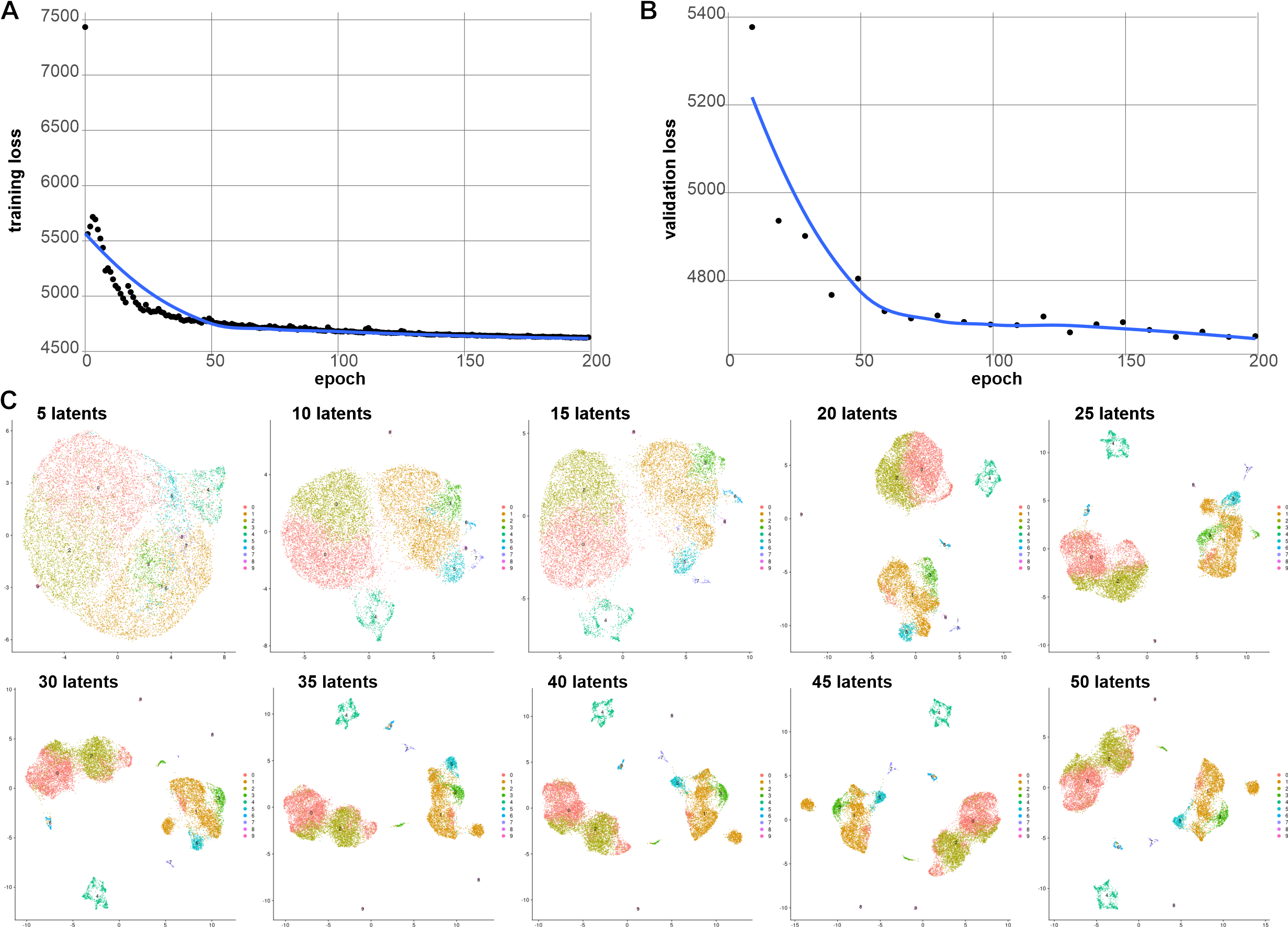

Supplement: Supplementary file 4 — Supplementary Figure 3 [file 41420_2025_2685_MOESM4_ESM.tif]

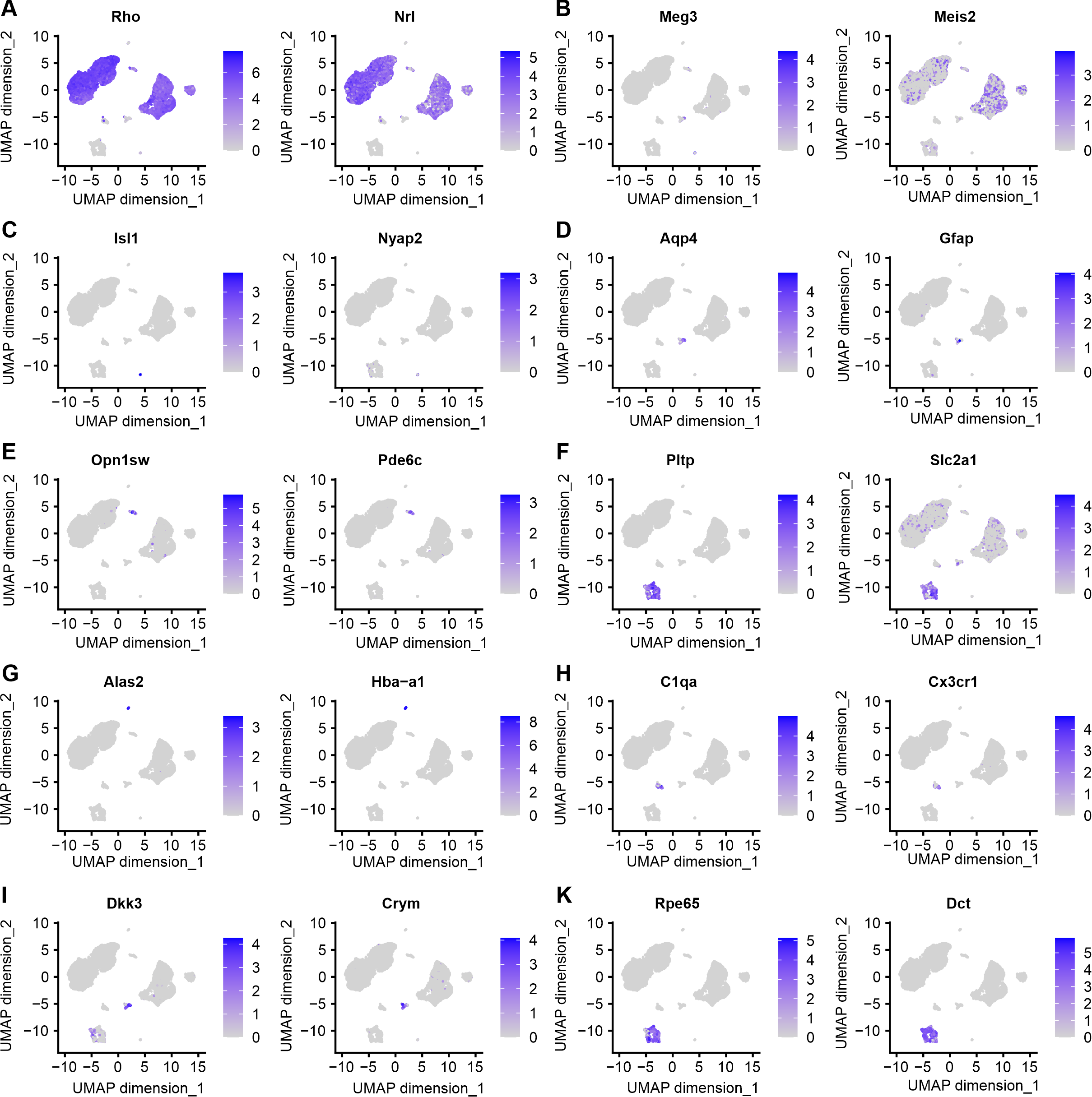

Supplement: Supplementary file 5 — Supplementary Figure 4 [file 41420_2025_2685_MOESM5_ESM.tif]
